# The Association Between Cognitive Functioning and Depression Severity: A Multiwave Longitudinal Remote Assessment Study

**DOI:** 10.1155/da/1509978

**Published:** 2025-02-26

**Authors:** Marcos Ross-Adelman, George Aalbers, Faith Matcham, Sara Simblett, Daniel Leightley, Sara Siddi, Josep M. Haro, Carolin Oetzmann, Vaibhav A. Narayan, Matthew Hotopf, Inez Myin-Germeys, Peter de Jonge, Femke Lamers, Brenda W. J. H. Penninx

**Affiliations:** ^1^Department of Psychiatry, Amsterdam UMC, Vrije Universiteit Amsterdam, Amsterdam, The Netherlands; ^2^Amsterdam Public Health, Mental Health Program, Amsterdam, The Netherlands; ^3^Department of Psychological Medicine, Institute of Psychiatry, Psychology and Neuroscience, King's College London, London, UK; ^4^School of Psychology, University of Sussex, Falmer, UK; ^5^School of Life Course and Population Sciences, Faculty of Life Sciences and Medicine, King's College London, London, UK; ^6^Parc Sanitari Sant Joan de Déu, Institut de Recerca San Joan de Déu (IRSJD), Sant Boi de Llobregat, Barcelona, Spain; ^7^Centro de Investigación Biomédica en Red en Salud Mental (CIBERSAM), Madrid, Spain; ^8^Department of Psychiatry, University of Oxford, Warneford Hospital, Oxford, UK; ^9^South London and Maudsley NHS Foundation Trust, London, UK; ^10^Department of Neurosciences, Center of Contextual Psychiatry, KU Leuven, Leuven, Belgium; ^11^Department of Developmental Psychology, University of Groningen, Groningen, The Netherlands

## Abstract

Cognitive difficulties are prevalent in depression and are linked to various negative life outcomes such as psychosocial impairment, absenteeism, lower chance of recovery or remission, and overall poor quality of life. Thus, assessing cognitive functioning over time is key to expanding our understanding of depression. Recent methodological advances and the ubiquity of smartphones enable remote assessment of cognitive functioning through smartphone-based tasks and surveys. However, the association of smartphone-based assessments of cognitive functioning to depression severity remains underexplored. Using a dedicated mobile application for assessing cognitive functioning (THINC-it), we investigate within- and between-person associations between performance-based (attention, working memory, processing speed, attention switching) and self-report measures of cognitive functioning with depression severity in 475 participants from the RADAR-MDD (Remote Assessment of Disease and Relapse-Major Depressive Disorder) cohort study (*t* = 2036 observations over an average of 14 months of follow-up). At the between-person level, we found stronger negative associations between the self-reported cognitive functioning measure and depression severity (*β* = −0.649, *p*  < 0.001) than between the performance-based measures and depression severity (*β*s = −0.220 to −0.349, *p*_s_  < 0.001). At the within-person level, we found negative associations between depression severity and the self-reported measure (*β* = −0.223, *p*  < 0.001), processing speed (*β* = −0.026, *p*=0.032) and attention (*β* = −0.037, *p*=0.003). These findings suggest that although THINC-it could adequately and remotely detect poorer cognitive performance in people with higher depressive symptoms, it was not capable of tracking within-person change over time. Nonetheless, repeatedly measuring self-reports of cognitive functioning showed more potential in tracking within-person changes in depression severity, underscoring their relevance for patient monitoring.

## 1. Introduction

Major depressive disorder (MDD) is a common condition, affecting about 350 million people worldwide per year (i.e., 4.7% of the population [1]). It is associated with a wide range of negative life outcomes, such as premature mortality and increased risk for physical diseases (e.g., cardiovascular disease, diabetes, and neurodegenerative disease), and ranks second among the leading causes of years lived with disability [2, 3]. Its symptomatology is pervasive, manifesting itself as a web of emotional, physiological, behavioral, and cognitive problems [4–6]. The present study focuses on the relationship between MDD severity and cognitive functioning.

Cognitive functioning encompasses capacities such as memory, reasoning, attention, verbal fluency, comprehension, problem-solving, and processing-speed [7, 8]. Many depressed individuals experience challenges in these areas, reporting memory deficits, thought disorganization, and concentration problems [9, 10]. These persistent cognitive difficulties have been linked to psychosocial impairment, absenteeism, poor quality of life, and a reduced chance of recovery or remission [10, 11]. Furthermore, even when treatment reduces depression symptomatology, cognitive problems often persist [12]. With this in mind, monitoring of cognitive functioning can offer valuable insights into the onset, course, and treatment response of depression [13, 14].

However, assessing cognitive functioning is challenging in clinical settings, often due to the resource and time-intensive nature of performance-based tasks and the difficulty with integrating their results with subjective self-reports, which are prone to bias [15, 16]. Moreover, traditional clinical/lab-based cognitive assessments fail to capture the contextual factors that influence performance in daily life [17].

With the growing development in the field of mobile health [18], our everyday devices (e.g., smartphones, tablets, and wearables) can be used to remotely assess cognitive functioning in daily life. This is advantageous for various reasons. First, remote data collection increases the ecological validity of the obtained data [19]. Second, assessments can be frequently repeated over time, providing a unique insight into the temporal dynamics of cognitive functioning, thus aiding in ongoing patient monitoring. Finally, the data can benefit clinician–patient communication, which ultimately facilitates more personalized healthcare [20].

Remote assessment tools have been employed to identify digital markers of cognitive functioning in persons with dementia [21, 22], schizophrenia [23], stroke [24], and in aging populations [25, 26]. However, research on the feasibility and specificity of these tools in depression is limited [27], underscoring the need for low-burden, reliable, and ecologically valid remote cognitive assessment tools for people living with depression.

One such tool is THINC-it [28], a digital tool specifically developed for MDD patients to measure cognitive functioning through a combination of self-report and performance-based measures. For the self-report measure, it uses the 5-item Perceived Deficit Questionnaire [29]. For the performance-based measures, it asks participants to complete four tasks that assess attention, working memory, processing speed, and attention switching—all domains frequently impaired in patients with depression. THINC-it requires ~10–15 min to complete, greatly reducing the usual 1–2 h administration time commonly seen in other cognitive test batteries [27, 28].

After illustrating its success in clinical settings [28, 30], one study incorporated this tool to remotely assess cognitive functioning in MDD patients: the RADAR-MDD (Remote Assessment of Disease and Relapse-Major Depressive Disorder) study, a multicenter longitudinal observational cohort study that followed over 600 people with a history of recurrent MDD to determine predictors of relapse over an average follow-up time of 18 months [31, 32].

Previous between-person analyses on the RADAR-MDD data examined the association between depression severity and cognitive functioning. Two main findings were observed [11]. First, all performance-based cognitive measures correlated moderately with each other but weakly correlated with the self-report measure. Second, people with persistent (multiple assessments of) cognitive difficulties, both self-reported and performance-based, tended to have higher depression severity throughout follow-up.

While these between-person insights are valuable, an important benefit of remote assessment tools is their capacity to frequently repeat measures over time. By doing so, they can capture within-person changes of any given health markers that can be used to monitor patients outside of the clinic [17, 18]. Therefore, to leverage cognitive functioning to monitor depression status over time, within-person associations need to be present as well. In other words, as depression severity improves or worsens, cognitive functioning should reflect a similar parallel change.

For the current paper, we expand earlier analyses of Matcham et al. [11] by applying multilevel modeling to investigate not only the between-person association between depression severity and cognitive functioning but also the longitudinal within-person association. We hypothesize that all individual THINC-it modules—both the self-report measure and performance-based tasks—are negatively associated with depression severity at the between- and within-person levels. Overall, the aim is to determine whether remotely assessed cognitive functioning can be used as a digital marker to monitor depression severity over time.

## 2. Materials and Methods

### 2.1. Sample

This paper presents a secondary analysis of the multicenter longitudinal observational RADAR-MDD cohort study [31]. RADAR-MDD enrolled 623 individuals from the Netherlands, Spain, and the United Kingdom who were aged over 18 years old and had a lifetime history of recurrent MDD (75% female, 79% white, *M*_age_ = 46.4 years, range [23, 84]). Participants engaged in regular self-report and performance-based assessments using a dedicated mobile application. Participants were followed up for an average of 18 months (range 11–24), with a median of 541 days [32]. Eligibility criteria included having met DSM-5 criteria for MDD, having experienced at least two episodes during their lifetime (with one occurring within the 2 years preceding study entry), participants' ability to provide informed consent, fluency in English, Dutch, Catalan, or Spanish, and willingness to use an Android smartphone throughout the study's duration. Exclusion criteria included a history of bipolar disorder, schizophrenia, MDD with psychotic features or schizoaffective disorder, a diagnosis of dementia, or a significant medical condition that could impede their ability to engage in typical daily activities for more than a 2-week period [31]. A full description of the RADAR-MDD sample, recruitment, data availability, and retention rates are available in Matcham et al. [32]. For the present study, a subset of 475 participants who provided assessments on depression severity and cognitive functioning were used. See Section 2.2 for the chosen assessments and Section 2.3 for the participant selection process.

### 2.2. Measures

#### 2.2.1. Cognitive Functioning

Cognitive functioning was measured using the digital tool THINC-it, a dedicated smartphone app for momentary cognitive assessments that takes 10–15 min to complete and can be self-administered by patients with minimal prior instruction [28]. For its use in RADAR-MDD, participants attended a training session where a member of the research team taught them how to use it. After this introduction, participants used THINC-it independently throughout the follow-up period, maintaining close contact with the research team. THINC-it covers five modules. Four performance-based tasks assess four domains of cognitive functioning: Spotter (the “Choice Reaction Time” test that measures attention via mean reaction time of correct responses), Symbol Check (the “1-back” test that measures working memory via number of correct responses), Codebreaker (the “Digit Symbol Substitution” test that measures processing speed via number of correct responses), and Trails (the “Trails Making Test B” that measures attention switching via completion time). For ease of interpretation, the four tasks were coded so that higher scores reflect higher cognitive performance. In this sense, correct answers in Codebreaker and Symbol Check already represent this, but mean reaction time from Spotter and completion time from Trails were recoded using reverse scoring. Additionally, Spotter and Trails had some extreme, though not outlier, scores in their lower bound, which is why the lower bound for these tasks was capped at the standard deviation minus three units. The fifth domain assessed the self-reported experience of cognitive functioning. This was done by asking participants to fill in the 5-item Perceived Deficits Questionnaire (PDQ-5; [29]). The PDQ-5 was then assessed as a sum score ranging from 0 to 20, where lower scores reflect a worse perception of one's cognitive functioning. The scores obtained in the tasks, alongside the PDQ-5 sum score, were individually used as the cognitive functioning variables for the analysis. Participants received a push notification sent to their smartphone to complete the THINC-it assessment every 6 weeks. All THINC-it modules have been validated against paper and pencil versions [28] and are sensitive to changes in cognitive functioning in adults with MDD [30, 33].

#### 2.2.2. Depression Severity

Depression severity was assessed with the 8-item Patient Health Questionnaire (PHQ-8; [34]). Participants received a push notification sent to their smartphone to complete the questionnaire every 2 weeks. The PHQ-8 items were summed, resulting in a composite score ranging from 0 to 24 where higher scores reflect greater severity.

#### 2.2.3. Demographic Factors

Age, gender, and years of education are known to be potential confounders of the association between cognitive function and mood [35, 36] and were therefore included in the analyses. Participants' age, gender, and years of education were collected at baseline.

### 2.3. Data Preprocessing

Data preprocessing was conducted using the *pandas* library (version 1.3.4) implemented in Python (version 3.9.7). The initial number of participants was 623, with a total of 13,478 PHQ-8 observations, 4148 PDQ-5 observations, 3734 Spotter observations, 3897 Symbol Check observations, 3757 Codebreaker observations, and 4059 Trails observations. We excluded participants who (a) prematurely withdrew from the study and asked for their data not to be used (*n* = 62), (b) did not complete all five THINC-it modules within a measurement occasion (*n* = 74), and (c) did not have PHQ-8 assessments that were temporally close to the THINC-it assessments (*n* = 12). Only PHQ-8 assessments that were provided within a maximum of ± 4 days apart from a complete set of THINC-it assessments were kept. In cases where more than one PHQ-8 assessment was within ±14 days from a complete set of THINC-it assessments, only the closest one was kept. Approximately 80% of PHQ-8 and THINC-it assessments were provided on the same day (Figure S1). We further excluded impossible scores for the analysis of Codebreaker (i.e., a negative number of correct answers, *n* = 69) and Trails (impossibly long completion times, e.g., >250,000 ms/4 min, *n* = 24) data, which also led to these observations being removed for the analysis in all the modules according to condition (b).

**Figure 1:**
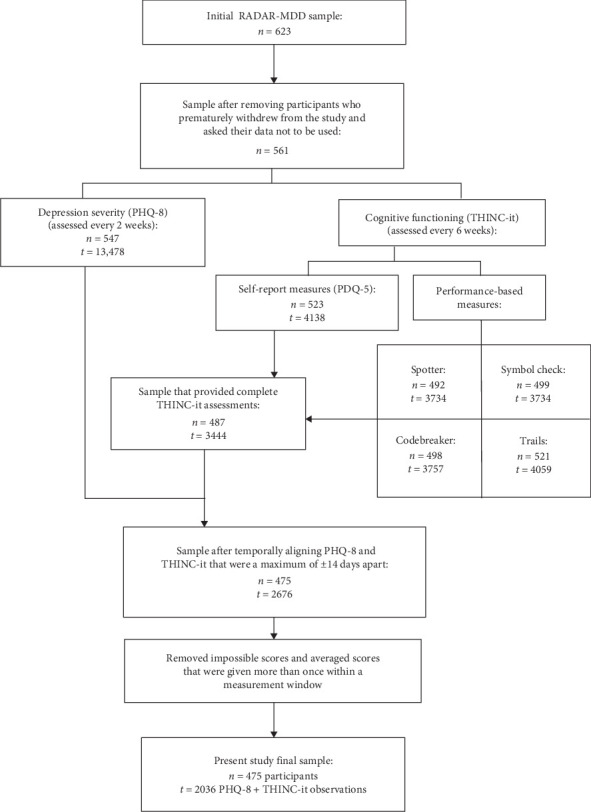
Flowchart of sample selection: *n* = participants and *t* = observations.

Because participants were enrolled at different times between 2017 and 2021, there were different start and end dates, as well as varying data contributions per participant. Thus, some data preprocessing was necessary to structure the data into a fixed measurements design. The result was a design that included 20 measurement occasions (baseline + 19) with a 6-week period in between each occasion. To achieve this fixed design, the following assumptions were considered: (a) Everyone's study period was determined by the participant who had been enrolled the longest. The longest period a participant had been enrolled was 798 days, making this number the study period for everyone (798 study period days divided by 6-week intervals = 19 occasions + 1 at baseline = 20 measurement occasions). (b) To temporally align all assessments, each participant's first assessment was considered their first day in the study. (c) “Measurement windows” were created to overcome the fact that participants did not provide data exactly every 6 weeks. Thus, all PHQ-8 + THINC-it assessments falling within the same 6-week window were considered as being part of the same measurement occasion. In general, participants followed protocol and provided data every 6 weeks (Figure S2). Sometimes participants provided a pair of PHQ-8 and THINC-it assessments more than once within a window. In these cases, the mean score of all assessments within a window was used to avoid discarding temporally aligned PHQ-8 + THINC-it assessments. Ultimately, the final sample size for the present study included 475 participants and 2036 temporally linked and complete PHQ-8 + THINC-it observations (refer to Figure 1 for a flowchart of participant selection) with an average follow-up time of ~418 days (≈ 14 months).

### 2.4. Data Analysis

We used the R software for the analysis (version 4.3.2) and its packages *lme4* (version 1.1-35.1) and *lmerTest* (version 3.1-3) to estimate two-level multilevel models (MLMs) using maximum likelihood estimation (MLE). An MLM was estimated for each of the five THINC-it modules as predictors and PHQ-8 as the outcome. A final MLM with the modules that showed a significant within-person association with PHQ-8 was also estimated. All code for preprocessing and data analysis is available in the OSF project URL (https://osf.io/5ajuv/).

**Figure 2:**
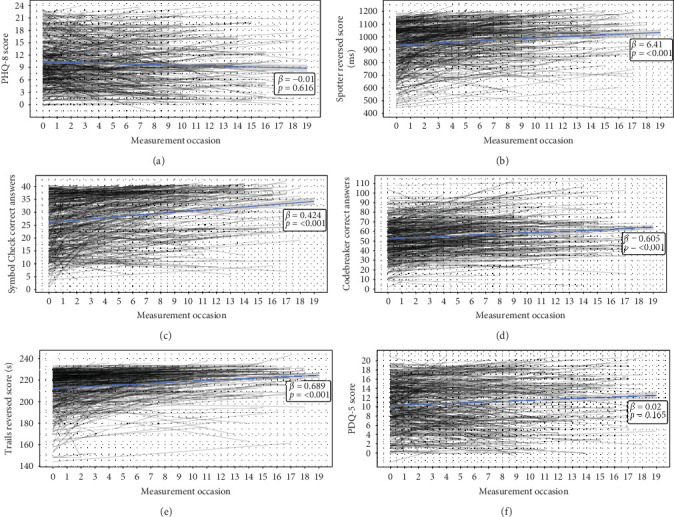
Time series plots of depression severity (PHQ-8) and the THINC-it cognitive functioning modules (Spotter, Symbol Check, Codebreaker, Trails, PDQ-5) for all participants (*n* = 475). Gray lines represent each individual trajectory. Blue lines represent the average linear trend of the whole sample's trajectory, with the effect of time shown by the *β* and *p*-values in each plot. Measurement occasions were every 6 weeks. (A) PHQ-8 time series plot; (B) Spotter time series plot; (C) Symbol Check time series plot; (D) Codebreaker time series plot; (E) Trails time series plot; (F) PDQ-5 time series plot. PDQ-5, 5-item Perceived Deficits Questionnaire; PHQ-8, 8-item Patient Health Questionnaire.

Because of the nested structure of the data (i.e., repeated measures within participants over time), two-level MLMs were estimated. This meant that we examined whether the relationship between cognitive functioning and depression severity differed between individuals (i.e., level-2) and within individuals over time (i.e., level-1). In order to obtain independent results for each level, it was necessary to first adjust the data, specifically the predictor variables (i.e., cognitive functioning scores). We person-mean centered the repeated measurements of the predictors [37], which separated the cognitive functioning scores into two components: a level-2 “trait” component (i.e., individual means that capture between-person differences) and a level-1 “state” component (i.e., person-centered means that capture within-person deviations from the mean over time). By entering the separate components of the cognitive functioning scores in the models (individual means and the deviations from the means), simultaneous and independent estimates of both the within-person and between-person effects of the predictor on the outcome were obtained [37]. MLMs were estimated in a stepwise manner, beginning with a model with no predictors (“null” or “intercept only” model) to identify the proportion of variance (i.e., the intraclass correlation or ICC) of the outcome (i.e., depression severity) at each level. We assumed there were random intercepts based on Figure 2A, which illustrates different baseline PHQ-8 scores across participants. We incrementally increased model complexity until reaching the best-fitting model. Deviance difference between models was used to assess improvements in model fit. Missing data were handled with the standard procedure for MLM which is listwise deletion of missing cases [38]. We reported standardized *β* coefficients to compare estimates across models. We standardized the variables to have a mean of 0 and a standard deviation of 1. We corrected for multiple testing by dividing the alpha by the total number of models (0.05/6 = 0.0083). Assumptions for MLM were also checked.

## 3. Results

### 3.1. Sample Characteristics and Descriptive Statistics

The final sample consisted of 475 participants (76.4% female, *M*_age_ = 50.8, range [23, 84], *M*_years of education_ = 16.4) with a total of 2036 complete PHQ-8 + THINC-it observations over a mean follow-up time of 14 months. On average, participants completed four PHQ-8 and THINC-it assessments (within 14 days of each other) according to the conditions set in Section 2. Baseline demographic information and descriptive statistics of the averaged scores of the measures can be found in Table 1. Time series plots showing how participants' answers and scores behaved over time can be found in Figure 2. Depressive symptom severity and self-reported cognitive functioning did not significantly change over time (Figure 2A,F), but performance-based cognitive performance did improve significantly, which may indicate learning effects over time (Figure 2B–E). Additionally, to quantify how much variance is attributable to differences between participants versus within-participant variability over time, the ICC for all variables was calculated. The ICCs ranged from 0.7 to 0.8 (0.7 for PHQ-8, 0.718 for Trails, 0.758 for Symbol Check, 0.771 for Spotter, 0.797 for PDQ-5, and 0.8 for Codebreaker).

### 3.2. The Association Between Cognitive Functioning and Depression Severity

MLMs were constructed to evaluate the association between cognitive functioning as the predictor and depression severity as the outcome over time. The random intercept null model—only including PHQ-8—yielded an ICC of 0.7, indicating that 70% of the variance in depression severity over time is at the between-person level, while 30% of the variance is at the within-person level.

#### 3.2.1. Performance-Based Cognitive Functioning and Depression Severity

For the association between the performance-based cognitive functioning modules of THINC-it and depression severity, MLMs with random intercepts for PHQ-8 were estimated. We assumed participants had significantly different baseline levels of depression severity (i.e., random intercepts) based on Figure 2A and statistically confirmed this (Table 2). Adding random slopes for cognitive performance and cross-level interactions did not increase explained variance. Thus, the best-performing models included the cognitive performance predictors at both levels and the demographic factors.

For the association between attention and depression severity, cognitive functioning at both the within-person (*β* = −0.037, SE = 0.013, *p*=0.003) and between-person levels (*β* = −0.302, SE = 0.047, *p*  < 0.001) had a significant negative association with depression severity. Similar results were found in the model testing the association between processing speed and depression severity (*β*_within-person_ = −0.026, SE = 0.012, *p*=0.032; *β*_between-person_ = −0.349, SE = 0.050, *p*  < 0.001). Regarding the association between working memory and depression severity, no association was found at the within-person level, and only the between-person cognitive functioning component showed a significant negative association (*β* = −0.220, SE = 0.049, *p*  < 0.001). Finally, when testing the association between attention switching and depression severity, again, no association was found at the within-person level, and only the between-person cognitive performance component showed a significant negative association (*β* = −0.269, SE = 0.048, *p*  < 0.001).

**Figure 3:**
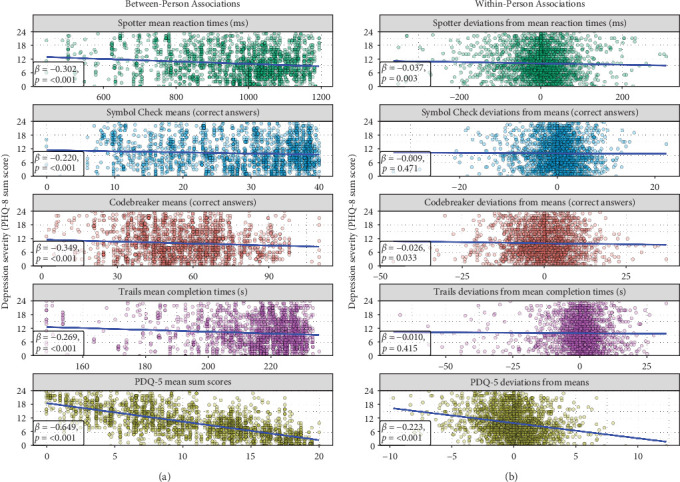
Panel plot illustrating the associations between depression severity (measured with the PHQ-8; shown as a common *y*-axis for all plots) and the cognitive functioning THINC-it modules. (A) Contains the associations at the between-person level and includes participants' mean scores for each of the cognitive functioning modules (*n* = 475). (B) Contains the associations at the within-person level and includes the deviations from the mean cognitive functioning scores (*t* = 2036). Blue lines represent the average linear trend, and their standardized estimates (*β*) and significance (*p*) are shown at the bottom left corner of each plot. Results have been adjusted for age, gender, and years of education.

Between-person level effects (i.e., differences between people's mean cognitive functioning scores) showed that individuals with lower scores in these tasks tended to have higher depression severity. The largest effect was found for processing speed, then attention, attention switching, and the smallest effect was found for working memory. At the within-person level, only in the attention and processing speed tasks were significant effects found. This means that, on average, higher levels of depression severity were linked to occasions when someone scored below their personal mean on attention and processing speed tasks. Results did not change when controlling for demographic factors. These results are illustrated in Figure 3. Overall, the explained variance of the models ranged from 9.1% to 12.5%, with most of the variance being explained by age and years of education (7.5%–10%). The explained within-person and between-person effects of the performance-based cognitive functioning predictors did not surpass 0.2% and 2.5% of explained variance, respectively. Therefore, the effect size of the observed associations was very small.

#### 3.2.2. Self-Reported Cognitive Functioning and Depression Severity

Self-reported cognitive functioning showed stronger associations with the PHQ-8 scores. As shown in Figure 3, predictors at both levels had a significant negative association with depression severity. At the within-person level, people with decreases in their mean PDQ-5 score tended to have higher depression severity (*β* = −0.223, SE = 0.014, *p*  < 0.001). At the between-person level, on average, individuals with higher PDQ-5 mean scores tended to have lower PHQ-8 mean scores compared to individuals with lower PDQ-5 mean scores (*β* = −0.649, SE = 0.028, *p*  < 0.001). Results did not change when controlling for demographic variables. Overall, the explained variance of the model was of 51.5%, of which 41.9% was explained at the between-person level, and 9.6% was explained at the within-person level.

#### 3.2.3. Combining Performance-Based and Self-Reported Cognitive Functioning Variables in One Model

To examine whether integrating information from multiple cognitive measures could improve depressive symptoms prediction, a final MLM with all THINC-it modules that showed a significant within-person association with PHQ-8 was estimated. This model showed significant between-person negative associations between depression severity and self-reported cognitive function (*β* = −0.610, SE = 0.029, *p*  < 0.001), processing speed (*β* = −0.104, SE = 0.040, *p*=0.010), and attention (*β* = −0.081, SE = 0.037, *p*=0.031). At the within-person level, only the self-report measure showed a significant negative association with depression severity (*β* = −0.230, SE = 0.011, *p*  < 0.001). The explained variance of the model was 51.1%. Thus, the within-person effect of the performance-based measures disappeared (Table S1). Moreover, combining cognitive functioning predictors did not improve the model's performance when compared to the model with only PDQ-5 as a predictor.

## 4. Discussion

This study examined the associations between self-reported and performance-based cognitive functioning and depression severity using remotely collected data via smartphones. Data were collected every 6 weeks over an average follow-up of 14 months. The goal was to explore these associations at both the between-person and within-person levels, with a special focus on within-person changes over time due to their relevance in the context of remote patient monitoring [19, 20]. At the between-person level, we found that people in our sample with worse self-reported and performance-based cognitive functioning tended to have higher depression severity. At the within-person level, higher depression severity was significantly associated with below the average scores on performance-based measures of attention and processing speed and on the self-report measure. Consistent with previous work, the self-report measure of cognitive functioning showed a stronger association with depression severity than performance-based measures [28]. Additionally, between-person associations were stronger than within-person associations.

The larger effect size for the self-report measure aligns with findings that people with depression often perceive themselves more negatively. Nonetheless, our results highlight the value of remote and repeated measures of self-reported cognitive functioning for monitoring depression. Specifically, deviations from the self-reported mean score are informative for indicating parallel changes in depression severity. One way to implement this in clinical practice could be to ask patients to repeatedly complete a questionnaire on cognitive functioning every few weeks. By establishing a personal self-report average, clinicians could monitor deviations from said average and use that information to enrich the conversation with patients (e.g., with visual aid [39]).

As previous research has shown, people with worse cognitive functioning generally experience higher depression severity [40]. This association, observed with the use of lab-based cognitive battery tests, was reflected in our between-person findings. This demonstrates that remote assessment tools can also capture this association outside of the lab. However, for mobile health, the within-person level is especially important. The utility of remote assessment tools lies in their capacity to capture within-person changes over time to find predictors of any given health outcome [18]. With this in mind, although the THINC-it tool has had success in clinical research settings [28, 30], the small effect sizes we found at the within-person level suggest that its smartphone-based use may not be adequate for remote monitoring of performance-based cognitive functioning, at least not with the assessment schedule used in our study. Various reasons might explain these small effect sizes. First, although previous analysis of the RADAR-MDD study showed that there were no apparent associations between the number of assessments participants provided and baseline depression severity, the THINC-it assessments were the least abundant out of all the available assessments in RADAR-MDD [32]. This could be associated with depression severity during the study where, if a participant was experiencing high levels of depression severity, completing THINC-it tasks may have become more challenging. Second, the “learning effect” (i.e., the tendency to perform better over time with repeated opportunities to practice the tasks [41]) could have influenced the associations. In our sample, performance-based measures showed a significant positive association with time (Figure 2B–E). Although this is a common problem when measuring performance-based cognitive functioning, the effect sizes for time were small (explained variances ranged from 3.1% to 4.7% depending on the task; refer to Table S2), which is expected according to the THINC-it user guide [42]. Third, depression severity scores were generally stable during the study period (Figure 2A). Many participants did not show large changes in depression severity over time, lowering the chances of finding longitudinal associations at the within-person level. Interestingly, McIntyre et al. [30] showed that THINC-it is sensitive to changes in cognitive function among patients taking antidepressants. This suggests that THINC-it could still be useful to remotely monitor patients when the potential to experience a change in depression severity is larger, such as during treatment. Finally, it could be that using THINC-it outside of a controlled lab setting led to a loss of sensitivity due to unforeseen daily life factors. RADAR-MDD is a naturalistic study, making it challenging to control for all the variables that could influence cognitive functioning in daily life, such as sleep, alcohol use, environmental noise, physical activity, and/or emotional stress [17, 42]. Despite these limitations, a strength of our study is that we used validated instruments in a population that is challenging to study. Additionally, since performance-based and self-reported cognitive functioning independently contribute to patient functioning [28], it is also valuable that we used the THINC-it tool due to its integration of both.

In sum, this study is unique in its focus on within-person associations between remotely assessed cognitive function and depression severity. It aligns with previous work aiming to detect digital markers of cognitive functioning in depression [43, 44]. Our results illustrate that self-reported cognitive functioning has stronger negative associations with depression severity than performance-based tasks. This challenges the assumption that clinic/lab-based performance tasks best capture cognitive capacities that are relevant for everyday life. Cognitive functioning in daily life is dynamic, contextual, and complex [17], and our results suggest that remotely assessed performance-based tasks may lack the expected sensitivity. If we were to focus on performance, then further improvement on remote assessment tools and/or an alternative approach is needed. For instance, passively logging behaviors like smartphone keystroke dynamics (e.g., speed, mistakes) that could act as digital markers for cognition could offer additional insight beyond the completion of tasks [45]. In practice, clinicians could use a combination of multimodal data sources (collected actively and passively) to monitor patients with a more comprehensive view of their health [18]. Additionally, the ability of remote assessment tools to repeatedly collect data can support the exploration of longitudinal and bidirectional associations between depression and cognition. For example, investigating whether changes in one precede changes in the other could enhance our understanding of the course of depression. Doing so is important because, while treatment can reduce depression symptomatology, incomplete remission is common, and patients often continue to experience cognitive problems even during remission [12]. This stresses the relevance cognitive functioning has in depression.

In conclusion, developing effective remote cognitive assessment tools is a necessary and exciting challenge for the field of mobile health. These tools could be used to monitor cognitive functioning in depression and provide a deeper understanding of their interplay over time.

## 5. Conclusions

Cognitive difficulties are a core aspect of depression symptomatology, and patients often continue to experience these difficulties even when other symptoms have subsided. Thus, monitoring cognitive functioning is key to advancing the treatment of depression. Remote assessment tools like smartphones offer the possibility to follow patients more closely in everyday life by obtaining digital markers of any given health outcome. By using remotely collected longitudinal data (over an average of 14 months), we aimed to determine whether smartphone-based assessments of cognitive functioning can be used as a digital marker to monitor depression severity. Our analysis focused on the differences between self-reported and performance-based cognitive functioning, as well as associations found between people and within people, with a special focus on the latter. We found that individuals with poorer cognitive functioning generally showed higher depression severity, both in self-reported and performance-based assessments. Performance-based measures were weakly associated with depression severity at the within-person level, highlighting a need for more effective remote cognitive performance assessment tools. Self-reported measures had a stronger negative association with depression severity at both levels of analysis, underscoring their importance in ongoing patient monitoring.

## Figures and Tables

**Table 1:** Descriptive statistics of the used measures (*n* = 475, *t* = 2036).

Variables	Mean (SD)	Range
Baseline demographic factors (*n* = 475)		
Female gender (%)	76.4%	
Age	50.8 (15.6)	23–84
Years of education	16.4 (6.6)	0–36

Averaged depression severity (*t* = 2036)		
PHQ-8 (score)	10.0 (6.1)	0–24

Cognitive functioning (THINC-it modules)		
Averaged self-report score (*t* = 2036)		
PDQ-5 (score)	10.8 (5.5)	0–20
Averaged performance-based scores (*t* = 2036)		
Spotter (ms)	616.9 (172.4)	347–1578
Symbol Check (number of correct responses)	28 (10)	0–40
Codebreaker (number of correct responses)	56 (20)	0–128
Trails (s)	29.2 (22.4)	14.9–235.9

*Note:* The cognitive functioning THINC-it modules assess attention (Spotter), working memory (Symbol check), processing speed (Codebreaker), and attention switching (Trails).

Abbreviations: PDQ-5, 5-item Perceived Deficits Questionnaire; PHQ-8, 8-item Patient Health Questionnaire.

**Table 2:** Standardized associations between cognitive functioning (THINC-it modules) and depression severity (PHQ-8).

Model	PDQ-5–PHQ-8 (self-reported cognitive performance)				Spotter–PHQ-8 (attention)				Symbol Check–PHQ-8 (working memory)				Codebreaker–PHQ-8 (processing speed)				Trails–PHQ-8 (attention switching)			
Fixed effects coefficients	Estimate (SE)	95% CI		*p*-Value	Estimate (SE)	95% CI		*p*-Value	Estimate (SE)	95% CI		*p*-Value	Estimate (SE)	95% CI		*p*-Value	Estimate (SE)	95% CI		*p*-Value
Mean/intercept	**0.598 (0.145)**	**[0.313, 0.883]**		**<**0.001^**∗∗∗**^	**1.554 (0.211)**	**[1.139, 1.968]**		**<**0.001^**∗∗∗**^	**1.466 (0.217)**	**[1.039, 1.892]**		**<**0.001^**∗∗∗**^	**1.571 (0.209)**	**[1.160, 1.982]**		**<**0.001^**∗∗∗**^	**1.319 (0.205)**	**[0.916, 1.722]**		**<**0.001^**∗∗∗**^
Time (occasion)	0.001 (0.003)	[−0.005, 0.006]		0.805	0.003 (0.003)	[−0.003, 0.010]		0.329	0.001 (0.003)	[−0.006, 0.008]		0.812	0.002 (0.003)	[−0.004, 0.009]		0.455	0.001 (0.003)	[−0.006, 0.007]		0.817
Cognitive functioning within-person component	**−0.223 (0.014)**	**[−0.251, −0.195]**		**<**0.001^**∗∗∗**^	**−0.037 (0.013)**	**[−0.062, −0.013]**		0.003^**∗∗**^	−0.009 (0.013)	[−0.034, 0.016]		0.471	**−0.026 (0.012)**	**[−0.051, −0.002]**		0.032^**∗**^	−0.010 (0.012)	[−0.034, 0.014]		0.415
Cognitive functioning between-person component	**−0.649 (0.028)**	**[−0.705, −0.594]**		**<**0.001^**∗∗∗**^	**−0.302 (0.047)**	**[−0.394, −0.210]**		**<**0.001^**∗∗∗**^	**−0.220 (0.049)**	**[−0.315, −0.124]**		**<**0.001^**∗∗∗**^	**−0.349 (0.050)**	**[−0.448, −0.250]**		**<**0.001^**∗∗∗**^	**−0.269 (0.048)**	**[−0.363, −0.176]**		**<**0.001^**∗∗∗**^
Age	**−0.004 (0.002)**	**[−0.007, 0.003]**		0.045^**∗**^	**−0.020 (0.003)**	**[−0.026, −0.014]**		**<**0.001^**∗∗∗**^	**−0.018 (0.003)**	**[−0.024, −0.012]**		**<**0.001^**∗∗∗**^	**−0.023 (0.003)**	**[−0.029, −0.017]**		**<**0.001^**∗∗∗**^	**−0.017 (0.003)**	**[−0.023, −0.012]**		**<**0.001^**∗∗∗**^
Gender (ref = male)	−0.093 (0.065)	[−0.221, 0.034]		0.150	0.012 (0.090)	[−0.166, 0.189]		0.898	0.022 (0.092)	[−0.159, 0.203]		0.814	0.093 (0.090)	[−0.084, 0.270]		0.304	0.053 (0.091)	[−0.127, 0.232]		0.566
Years of education	**−0.016 (0.005)**	**[−0.026, −0.006]**		0.002^**∗∗**^	**−0.029 (0.007)**	**[−0.042, −0.015]**		**<**0.001^**∗∗∗**^	**−0.031 (0.007)**	**[−0.045, −0.017]**		**<**0.001^**∗∗∗**^	**−0.025 (0.007)**	**[−0.039, −0.011]**		0.001^**∗∗**^	**−0.024 (0.007)**	**[−0.038, −0.010]**		0.001^**∗∗**^

**Random effects coefficients**	**Estimate (SD)**				**Estimate (SD)**				**Estimate (SD)**				**Estimate (SD)**				**Estimate (SD)**			

Level-1 error term	0.199 (0.446)				0.291 (0.539)				0.293 (0.541)				0.291 (0.540)				0.292 (0.540)			
Level-2 error term	0.274 (0.523)				0.570 (0.755)				0.594 (0.771)				0.562 (0.750)				0.583 (0.763)			

**Explained variance (*R*** ^**2**^ **)**	**Level**		**Total**		**Level**		**Total**		**Level**		**Total**		**Level**		**Total**		**Level**		**Total**	

*R* ^2^ Level-1	0.321		0.096		0.006		0.002		0.000		0.000		0.004		0.001		0.000		0.000	
*R* ^2^ Level-2	0.599		0.419		0.165		0.116		0.130		0.091		0.176		0.123		0.146		0.102	
Total *R*^2^	0.515 (51.5%)				0.118 (11.8%)				0.091 (9.1%)				0.124 (12.4%)				0.102 (10.2%)			

*Note:* Level-1 is the within-person level. Level-2 is the between-person level. Significant associations are in bold.

Abbreviations: PDQ-5, 5-item Perceived Deficits Questionnaire; PHQ-8, 8-item Patient Health Questionnaire.

*⁣* ^*∗*^ *p*  < 0.05.

*⁣* ^*∗∗*^ *p*  < 0.0083 (corrected alpha for multiple comparisons—*α*/6).

*⁣* ^*∗∗∗*^ *p*  < 0.001.

## Data Availability

The data needed to replicate the analyses in this paper are not publicly accessible. Nonetheless, the code required for replication, alongside supporting information, is available on the OSF project URL (https://osf.io/5ajuv/).

## Supporting Information

Supporting Information We have included Figures S1 and S2 to support some statements we made in Section 2.3. While preprocessing the data, our goal was to first link PHQ-8 and THINC-it assessments that were close in time to ensure that the associations we found between them made sense, temporally speaking. We decided on a maximum of ±14 day difference between PHQ-8 and THINC-it assessments because a greater interval would be too large, thus complicating the interpretation of the results. Figure S1 illustrates that approximately 80% of the linked PHQ-8 and THINC-it assessments were provided on the same day. This indicates that most of the times, when participants completed a PHQ-8 assessment, they also completed the THINC-it assessments on the same day. We think this is a relevant piece of information because it supports the temporal aspect of the relationship between depression severity and cognitive functioning. Additionally, given that RADAR-MDD was a naturalistic study, we wondered whether participants followed the protocol of providing THINC-it assessments every 6 weeks. While preparing the data, we had already noticed that participants did not always follow protocol, so we wanted to check how often this happened. Figure S2 is the result of that check. We found that most of the times, assessments were provided every 6 weeks. Finally, we included two supporting tables. Table S1 was added because we wanted to examine whether integrating information from multiple cognitive measures could improve depressive symptom prediction. We estimated a final multilevel model with the THINC-it modules that had previously shown a significant within-person association with PHQ-8. We thought the idea of combining performance-based and self-reported cognitive functioning variables as predictors in one model was a good idea to test, which is why we included it in Section 3. However, depression severity prediction was not improved, so we decided to include this table as supporting information. Table S2 was added to clarify Reviewer 1's comment on the learning effect we found with the performance-based cognitive functioning measures. The THINC-it guide states that a small learning effect is to be expected, which is what we found. To show this, we included the table.
