# Supplementary material for: The Association Between Cognitive Functioning and Depression Severity: A Multiwave Longitudinal Remote Assessment Study
Source: Depress Anxiety. 2025 Feb 26;2025:1509978. doi: 10.1155/da/1509978 (PMC11918956; doi:10.1155/da/1509978)
Supplement: Supporting Information — We have included Figures S1 and S2 to support some statements we made in Section 2.3. While preprocessing the data, our goal was to first link PHQ-8 and THINC-it assessments that were close in time to ensure that the associations we found between them made sense, temporally speaking. We decided on a maximum of ±14 day difference between PHQ-8 and THINC-it assessments because a greater interval would be too large, thus complicating the interpretation of the results. Figure S1 illustrates that approximately 80% of the linked PHQ-8 and THINC-it assessments were provided on the same day. This indicates that most of the times, when participants completed a PHQ-8 assessment, they also completed the THINC-it assessments on the same day. We think this is a relevant piece of information because it supports the temporal aspect of the relationship between depression severity and cognitive functioning. Additionally, given that RADAR-MDD was a naturalistic study, we wondered whether participants followed the protocol of providing THINC-it assessments every 6 weeks. While preparing the data, we had already noticed that participants did not always follow protocol, so we wanted to check how often this happened. Figure S2 is the result of that check. We found that most of the times, assessments were provided every 6 weeks. Finally, we included two supporting tables. Table S1 was added because we wanted to examine whether integrating information from multiple cognitive measures could improve depressive symptom prediction. We estimated a final multilevel model with the THINC-it modules that had previously shown a significant within-person association with PHQ-8. We thought the idea of combining performance-based and self-reported cognitive functioning variables as predictors in one model was a good idea to test, which is why we included it in Section 3. However, depression severity prediction was not improved, so we decided to include this table as supporting information. Table S2 was [file 1509978.f1.docx]

##
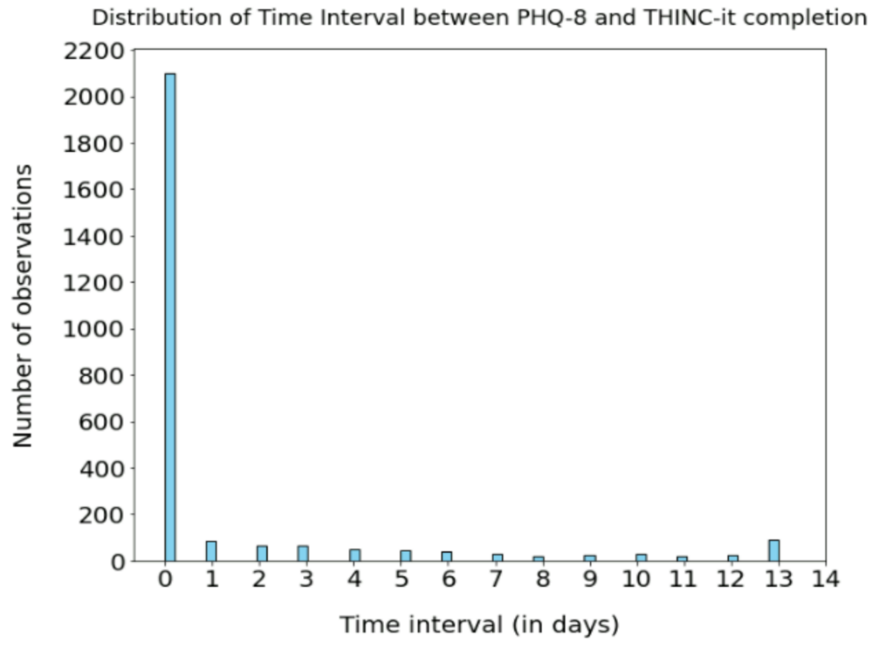
Supplementary Materials

**Supplementary Figure S1.** Distribution of time interval between PHQ-8 and THINC-it^®^ completion. Approximately 80% of depression severity and cognitive functioning assessments were completed on the same day.


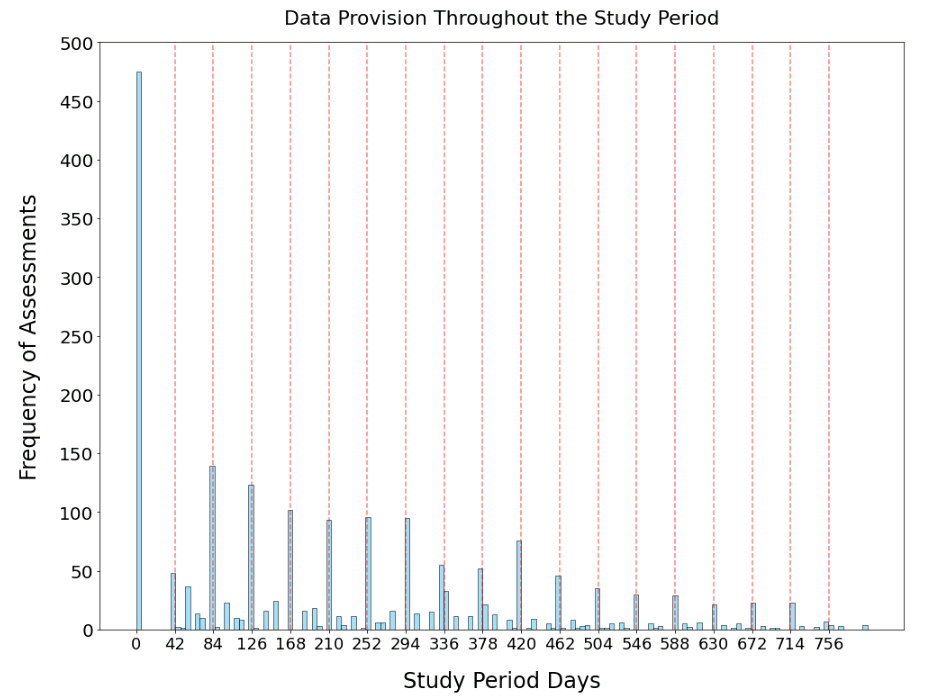


**Supplementary Figure S2**. Distribution of data points throughout the study period. Red dotted lines indicate the beginning of a new measurement window as explained in the ‘Data preprocessing’ part of the ‘Methods’ section. In general, most assessments are provided data every 42 days/6 weeks.

| Model | PDQ-5-Spotter-Codebreaker – PHQ-8  (Self-report + attention + processing speed) | | | |
| --- | --- | --- | --- | --- |
|  |  |  |  |  |
| Fixed effects coefficients | Estimate (S.E.) | 95% CI | | p-value |
| Mean/Intercept | **0.878 (0.157)** | **[0.570, 1.185]** | | **<0.001***** |
| Time (occasion) | 0.004 (0.003) | [-0.001, 0.010] | | 0.132 |
| PDQ-5 within-person component | **-0.230 (0.011)** | **[-0.251, -0.210]** | | **<0.001***** |
| PDQ-5 between-person component | **-0.610 (0.029)** | **[-0.667, -0.553]** | | **<0.001***** |
| Spotter within-person component | -0.007 (0.011) | [-0.029, 0.015] | | 0.507 |
| Spotter between-person component | -0.081 (0.037) | [-0.154, -0.008] | | 0.031 |
| Codebreaker within-person component | -0.017 (0.011) | [-0.039, 0.004] | | 0.113 |
| Codebreaker between-person component | -0.104 (0.040) | [-0.183, -0.025] | | 0.010 |
| Age | **-0.011 (0.002)** | **[-0.016, -0.006]** | | **<0.001***** |
| Gender (ref=male) | -0.064 (0.064) | [-0.191, 0.062] | | 0.318 |
| Years of education | -0.012 (0.005) | [-0.022, -0.002] | | 0.018 |
| Random effects coefficients | Estimate (S.D.) | | | |
| Level-1 error term | 0.222 (0.471) | | | |
| Level-2 level error term | 0.254 (0.504) | | | |
| Explained Variance (R^2^) | Level | | Total | |
| R^2^ Level-1 | 0.241 | | 0.072 | |
| R^2^ Level-2 | 0.627 | | 0.439 | |
| Total R^2^ | 0.511 (51.1%) | | | |

**Supplementary Table S1**. Standardized associations between the three cognitive functioning modules (self-report, attention, processing speed) that showed a significant negative association with depression severity (PHQ-8). Level-1 is the within-person level. Level-2 is the between-person level. Significant associations are in bold.

*p <0.05, **p<0.0083 (corrected alpha for multiple comparisons – α/6), ***p<0.001.

| Cognitive Functioning | PDQ-5  (Self-reported cognitive functioning) | | Spotter  (Attention) | | Symbol Check  (Working memory) | | Codebreaker  (Processing speed) | | Trails  (Attention switching) | |
| --- | --- | --- | --- | --- | --- | --- | --- | --- | --- | --- |
| Fixed effects coefficients | Estimate (S.E.) | p-value | Estimate (S.E.) | p-value | Estimate (S.E.) | p-value | Estimate (S.E.) | p-value | Estimate (S.E.) | p-value |
| Mean/Intercept | **10.280 (0.238)** | **<0.001***** | **919.62 (7.565)** | **<0.001***** | **24.880 (0.453)** | **<0.001***** | **50.600 (0.886)** | **<0.001***** | **210.308 (0.803)** | **<0.001***** |
| Time (occasion) | 0.020 (0.015) | 0.165 | **6.410 (0.468)** | **<0.001***** | **0.424 (0.030)** | **<0.001***** | **0.605 (0.053)** | **<0.001***** | **0.689 (0.058)** | **<0.001***** |
| Explained variance (R^2^) | Level | Total | Level | Total | Level | Total | Level | Total | Level | Total |
| R^2^ Level-1 | 0.000 | 0.000 | 0.102 | 0.022 | 0.106 | 0.026 | 0.069 | 0.014 | 0.075 | 0.021 |
| R^2^ Level-2 | 0.003 | 0.003 | 0.014 | 0.011 | 0.028 | 0.021 | 0.022 | 0.017 | 0.017 | 0.012 |
| Total R^2^ explained by time | 0.003 (0.3%) | | 0.033 (3.3%) | | 0.047 (4.7%) | | 0.031 (3.1%) | | 0.033 (3.3%) | |
| Interpretation | PDQ-5 scores do not significantly increase over time | | On average, with every new occasion, participants improve in their Spotter scores by 6.41 milliseconds | | On average, with every new occasion, participants improve their Symbol Check scores by 0.42 answers | | On average, with every new occasion, participants improve their Codebreaker scores by 0.6 answers | | On average, with every new occasion, participants improve in their Trails scores by 0.69 seconds | |

**Supplementary Table S2**. Associations between time and the THINC-it^®^ modules to assess the effect size of the learning effect (i.e., the tendency to perform better over time with repeated opportunities to practice the tasks). Measurement occasion was the predictor and the cognitive functioning variables were the outcomes. Level-1 is the within-person level. Level-2 is the between-person level. Significant associations are in bold.
